# Correction: The leptomeninges as a critical organ for normal CNS development and function: First patient and public involved systematic review of arachnoiditis (chronic meningitis)

**DOI:** 10.1371/journal.pone.0306765

**Published:** 2024-07-03

**Authors:** Carol S. Palackdharry, Stephanie Wottrich, Erin Dienes, Mohamad Bydon, Michael P. Steinmetz, Vincent C. Traynelis

The first author’s name is spelled incorrectly. The correct name is: Carol S. Palackdharry. The correct citation is: Palackdharry CS, Wottrich S, Dienes E, Bydon M, Steinmetz MP, Traynelis VC (2022) The leptomeninges as a critical organ for normal CNS development and function: First patient and public involved systematic review of arachnoiditis (chronic meningitis). PLoS ONE 17(9): e0274634. https://doi.org/10.1371/journal.pone.0274634
