# Supplementary material for: The leptomeninges as a critical organ for normal CNS development and function: First patient and public involved systematic review of arachnoiditis (chronic meningitis)
Source: PLoS One. 2022 Sep 30;17(9):e0274634. doi: 10.1371/journal.pone.0274634 (PMC9524710; doi:10.1371/journal.pone.0274634)
Supplement: Attachment — Submitted filename: Response to reviewers V1.docx [file pone.0274634.s002.docx]

7/8/22

**To**: Dr. Panagiotis Kerezoudis and the PLOS Editorial Team

**From**: Drs. Palackdharry, Wottrich, Dienes, Bydon, Steinmetz, and Traynelis

**RE**: **Response to reviewers** for PLOS ONE: PONE-D-22-02599 - [EMID:90cbb9b5ed794546]

**Attached please find [1] a track change document which highlights all changes made to the original manuscript labelled “Revised manuscript with track changes,” and [2] a clean, unmarked revised paper labelled “manuscript.”**

Below is a list of revisions and/or rebuttals without revisions:

1. Throughout the abstract, paper, and tables, the total number of study and DLMs patients has increased 61 patients to 133261 and 12721, respectively. This is due to an extraction error in the spinal cord stimulation patients discovered in writing the new section [North published 2 papers in the same year and the case report was accidentally extracted instead of the study with 61 patients]. The resulting numbers further support conclusions, but do not change overall study percentages due to the large number of patients in the study.
2. The second sentence of the conclusion of the abstract was rearranged since the word “function” was repeated.
3. From Dr. Panagiotis Kerezoudis, Academic Editor
   a. The abbreviation “PPI” was spelled out in the title
   b. Author bylines and affiliations were corrected according to the PLOS formatting guidelines
   c. Author symbol legend was corrected according to the PLOS formatting guidelines
   d. All figures were remade in high resolution 600 dpi .tiff, uploaded to the PACE Tool, which converted down to 300 dpi automatically and with the .tif extension. These did not give good resolution at smaller sizes, so we are uploading the new high resolution 600 dpi.tiff files
   e. Tables 5-8 were reformatted to fit the standard layout
   f. Tables and captions are no longer embedded in the text. All tables with legends were moved to the end of the text, prior to references, and the reference numbers appropriately changed.
   g. Figure legends were moved to after the references.
   h. Figure 2 has been referenced [as all pictures were published in a journal article]
   i. Competing interests have been shared. This does not alter our adherence to PLOS ONE policies on sharing data and materials.
   j. Competing interests have been updated and there are no changes
   k. The data was presented as a podium poster presentation AANS in July 2021. The poster was peer-reviewed but not published as part of the conference proceedings. Therefore, no work constitutes dual publication, and all data should be included in the manuscript.
   l. All data used for the manuscript has been made available in Table 5. We will change the data availability statement to reflect that all relevant data is available within the manuscript.
   m. We are unsure of the source of the comment regarding inclusion of Bewick et al [5]. We do not have that citation in our Zotero database or cited anywhere in the article. There is no reference to this citation in our document.
   n. A caption for Figure 5 has been provided after the references.
4. **Response to reviewers # 1 & 2**: All figures were changed to high resolution [300 dpi] using the PACE tool after retaking image shots. We thank the reviewers for their time and comments.
5. **Response to reviewer #3:**
   a. We believe the title of the article supports the conclusion that the LM are a distinct organ. There is a large body of animal and bench research supporting this concept. Our exhaustive PSR in humans not only supports our conclusion but opens a new field in neuroscience to prevent infectious, autoimmune, toxic, and iatrogenic damage that leads to fibrosis of the critical structures that traverse the SAS and support remodeling of the CNS throughout life.
   b. We agree a quantitative analysis of the broad range of data is not possible, which is why we did not attempt one. Statistical analysis is possible with certain portions of the data, which have already been performed and reported. We have added additional studies not included in those analyses.
   c. We do not believe our conclusions are subjective, but solidly based on our data as well as a wealth of pathologic and pre-clinical data.
   d. The broken links to the published PSR have been fixed.
   e. We have provided substantial evidence that author-industry relationships bias published literature and legal data increase knowledge of possible harms. When devices or techniques cause harm, settlements often prevent publication of those harms and thus the literature can overestimate benefit. These concepts are discussed and referenced in the article.
   f. We agree the field of medicine has changed over the nearly 200-year span of this PSR, thus we did not attempt make conclusions impacted by changes in imaging and surgical techniques. Going forward, with the information we have integrated, it will hopefully be easier to create and share innovations.
6. **Response to reviewer #4:** we thank this reviewer for their careful review and helpful comments.
   a. PPI was spelled out in the title and all noted typos were fixed.
   b. We added details of how the AE consistent with DLMs were adjudicated to come up with the resulting number of 632 added events in the “iatrogenic DLM” section
   c. Table width was fixed for tables 5-8
   d. Denominators were changed to be consistent throughout the manuscript. The new denominator is 12721. There were 50 patients with SCS implanted for “lumbar meningeal fibrosis” which were added when we re-extracted all 10 SCS articles.
   e. We added in the immunomodulators, and anti-infective agents used to treat compressive abscesses.
   f. We answered the question of AE observed in non-DM metformin users. FYI, it was well tolerated, as evidenced by a 2022 review now cited in the discussion [no data were included as it was out of the cutoff range but used for discussion].
   g. We added in the proposed mechanisms for rhBMP-2 DLMs in the molecular biology section. There is substantial information on many other AE associated with this BMP2 [non- DLMs] which are in the references. We added a sentence about reduced doses in the iatrogenic section, as well as discuss the role of multiple surgeries.
   h. We are immensely grateful reviewer 4 pointed out we left out the section on SCS, despite specifically planning for it. We agree it is a critical issue and added in a section under “treatment,” as well as rearranged the treatments from practical at this moment to theoretical and unproven.
